# Structural, functional, and metabolic signatures of postpartum depression: A systematic review

**DOI:** 10.3389/fpsyt.2022.1044995

**Published:** 2022-11-16

**Authors:** Anna Horáková, Hana Němcová, Pavel Mohr, Antonin Sebela

**Affiliations:** ^1^Center of Perinatal Mental Health, National Institute of Mental Health, Klecany, Czechia; ^2^Department of Psychology, Faculty of Arts, Charles University, Prague, Czechia; ^3^Department of Psychiatry and Medical Psychology, Third Faculty of Medicine, Charles University, Prague, Czechia; ^4^Clinical Center, National Institute of Mental Health, Klecany, Czechia

**Keywords:** postpartum depression, neuroimaging, fMRI, magnetic resonance spectroscopy, perinatal depression, structural MRI, magnetic resonance imaging

## Abstract

**Objective:**

Postpartum depression (PPD) is a serious condition with debilitating consequences for the mother, offspring, and the whole family. The scope of negative outcomes of PPD highlights the need to specify effective diagnostics and treatment which might differ from major depressive disorder (MDD). In order to improve our clinical care, we need to better understand the underlying neuropathological mechanisms of PPD. Therefore, we conducted a systematic review of published neuroimaging studies assessing functional, structural, and metabolic correlates of PPD.

**Methods:**

Relevant papers were identified using a search code for English-written studies in the PubMed, Scopus, and Web of Science databases published by March 2022. Included were studies with structural magnetic resonance imaging, functional magnetic resonance imaging, both resting-state and task-related, magnetic resonance spectroscopy, or positron emission tomography. The findings were analyzed to assess signatures in PPD-diagnosed women compared to healthy controls. The review protocol was registered in PROSPERO (CRD42022313794).

**Results:**

The total of 3,368 references were initially identified. After the removal of duplicates and non-applicable papers, the search yielded 74 full-text studies assessed for eligibility. Of them, 26 met the inclusion criteria and their findings were analyzed and synthesized. The results showed consistent functional, structural, and metabolic changes in the default mode network and the salient network in women with PPD. During emotion-related tasks, PPD was associated with changes in the corticolimbic system activity, especially the amygdala.

**Discussion:**

This review offers a comprehensive summary of neuroimaging signatures in PPD-diagnosed women. It indicates the brain regions and networks which show functional, structural, and metabolic changes. Our findings offer better understanding of the nature of PPD, which clearly copies some features of MDD, while differs in others.

## Introduction

Due to the physiological changes, disturbances in a sleep/wake cycle, and the number of accumulated psychosocial stressful events, perinatal and postnatal periods are challenging phases for women’s mental health. As a result, postpartum depression (PPD) may develop or exacerbate in vulnerable women with the estimated prevalence of 17%, which is higher than in any other period of women’s life (1).

Households affected by PPD incurred 22% higher mean total all-cause medical and pharmaceutical spending than unaffected matched controls (2). Furthermore, PPD reduces mothers’ quality of life (3), impairs their social relationships (4), and it can be a life-threatening condition since it is associated with increased risk of self-harm and suicidal ideations (5). PPD in mothers can diminish their ability to read and response to social and affective clues of the infant. Consequently, insecurities in the mother-child bonding create a challenging environment for the child’s growth which can have adverse effects on motor (6), cognitive (7), language (8), and social and emotional (9) development early in life. The scope of negative outcomes of PPD highlights the need to adjust effective treatment. Ideally, the optimum option how to minimize the potential debilitating consequences of PPD would be prevention and early psychosocial interventions (10). Better understanding of the neuropathological mechanisms of PPD might help to design more effective preventive and therapeutic strategies.

While the negative psychosocial impact of PPD is extensively covered in literature, the neural mechanisms behind the curtain of its pathogenesis are to be yet explained in a greater detail. Imaging techniques have been of crucial importance to help us understand the pathogenesis of psychiatric disorders, including major depressive disorder (MDD). However, there are only few imaging studies focusing specifically on PPD. So far, most of them focused either on neural response to affective stimuli or on brain metabolism changes in PPD. Duan et al. (11) in their review took a closer look into the fMRI emotional task results and identified the amygdala as the main region of interest, with the most significant changes in PPD.

The perinatal period alone is associated with substantial changes in the brain connectivity and structural integrity in healthy mothers, as their brains undergo the process of adaptation to the maternal caregiving (12, 13). Considering these essential transformations, it is of interest to examine whether the PPD brain features differ from those observed in a non-depressed peripartum mother. Furthermore, while some characteristics of PPD are identical to MDD (e.g., general symptoms such as low mood, sleep disturbances, or genetic underpinnings), other are more specific for PPD (e.g., extensive worries about baby health and life, mood-destabilizing effects of reproductive hormones exposure, estrogen-dependent epigenetic changes, and perinatally related psychosocial stressors) (14). These features suggest that PPD might be a distinct depression subtype with unique neural mechanisms.

Since 2017, when the review by Duan et al. (11) was published, numerous new studies investigating neurobiology of PPD have emerged. Our objective was to review the updated and current body of knowledge of the PPD signatures. Due to the fact that the majority of recent studies investigated the PPD-related brain changes in a non-active self-referential state, our special focus was on the resting state paradigm.

To examine the neural features of PPD would allow us (1) to detect functional, structural, and metabolic changes in mothers with PPD in comparison to healthy mothers, (2) to outline and discuss the functional, structural, and metabolic differences between PPD and MDD. The secondary objective was to discuss the results within the context of the brain functional networks: the default mode network (DMN), the salience network (SN), the cognitive control network (CNN), and the corticolimbic system as the most investigated circuits in the MDD research (15).

## Methods

The study protocol was registered in PROSPERO (CRD42022313794) under the title: ‘‘Structural, functional, and metabolic signatures of postpartum depression.’’ Screening of citations, full text review, assessment of risk bias and extraction of studies characteristics and outcomes in this systematic review were performed in the Covidence software platform^1^.

### Search strategy

We searched systematically the PubMed, Scopus, and Web of Science databases for English written articles published by the end of March 2022, without bottom time limitation. The search code was identical to the one used originally by Duan et al. (11), so the data would be methodologically comparable. Since our focus was on the postpartum depression only, the articles examining other types of peripartum depression were excluded from further analysis. In all databases, the following search terms were applied: (“diffusion imaging” or “brain mapping” or “brain morphology” or “connectome” or “dti” or “fmri” or “functional mri” or “functional magnetic resonance” or “functional neuroimaging” or “diffusion imaging” or “diffusion tensor imaging” or “functional neuroimaging” or “functional connectivity” or “magnetic resonance imaging” or “mri” or “magnetic resonance spectroscopy” or “mrs” or “neuroimaging” or “PET” or “structural mri” or “tomography” or “volumetric based morphometry” or “volume positron emission” or “volume based morphometry”) AND (“pregnancy” or “antepartum” or “perinatal” or “motherhood” or “postpartum” or “maternal” or “antenatal” or “postnatal” or “prepartum” or “peripartum” or “mothers”) AND (“depression” or “depressive”).

### Eligibility criteria

For data synthesis, the studies were grouped according to the imaging techniques used (i.e., functional, structural, or metabolic). For functional magnetic resonance imaging (fMRI), studies were then assigned to either resting-state (rs) or task-related group. Based on the character of the task, participants in the task-related group were further classified into the emotional-task paradigm group or reward-task paradigm group. No additional type of tasks in the PPD imaging studies has been identified in our search.

The inclusion criteria were: cohort or cross-sectional PPD studies using structural magnetic resonance imaging (MRI), functional magnetic resonance imaging (fMRI), magnetic resonance spectroscopy (MRS), or positron emission tomography (PET) to assess signatures of PPD; PPD in participants was determined by a structured clinical interview or other standardized diagnostic methods; study population consisted of a group of women diagnosed with PPD up to 12 months after the childbirth and the control group consisted of women without PPD up to 12 months after the childbirth.

The exclusion criteria were: meta-analyses, systematic or non-systematic reviews; the use of non-validated method to diagnose PPD; time of testing exceeded 12 months after the childbirth; the study group included bipolar patients or any other psychiatric comorbidity except for anxiety disorders; the study did not have a control group.

Other potential confounding factors were taken into consideration without being a reason for the exclusion: medication status; comorbid anxiety disorder; first-onset or previous history of depression; socioeconomic status; education level; and number of previous pregnancies. If relevant, their potential confounding impact was discussed in the results.

### Risk of bias assessment

For the assessment of risk of bias, we used quality assessment checklist adapted for neuroimaging studies evaluation, specifically designed for the purpose of the current review. Two authors (AH and HN) independently assessed the quality of studies and agreement between them was required in order to rate the final score. The studies were assessed with 14 items (see Supplementary material 1) with the overall score range from 0 (very low quality) to 14 (very high quality).

### Study selection and data extraction

After the automatic duplicate elimination in the Covidence software, two authors (AH and HN) independently screened the titles and abstracts of all references identified by the search strategy, potential conflicts were resolved by a third author (AS). Subsequently, the full text review screening has been performed independently by two authors (AH and HN) and potential conflicts were resolved by a third author (AS). Data extraction (study ID; first author and year of publication; methods; paradigm; sample size; evaluation of depressive symptoms; time of testing after the childbirth; findings of neural features; coordinates if available; medication; comorbidity) was then performed and the quality assessment was completed for each study independently by two authors (AH and HN). A consensus between the two authors was required for the final inclusion of a study in the systematic review sample.

### Analysis and synthesis of the results

Since the characteristics varied highly across the studies, we could not perform originally planned meta-analysis to compare data statistically. Thus, for a systematic comparison of the functional, structural, and metabolic correlates of women with PPD and without PPD, a narrative analysis with the main outcome of changes in the brain regions, networks, and metabolic signatures of PPD was used.

## Results

**FIGURE 1:**
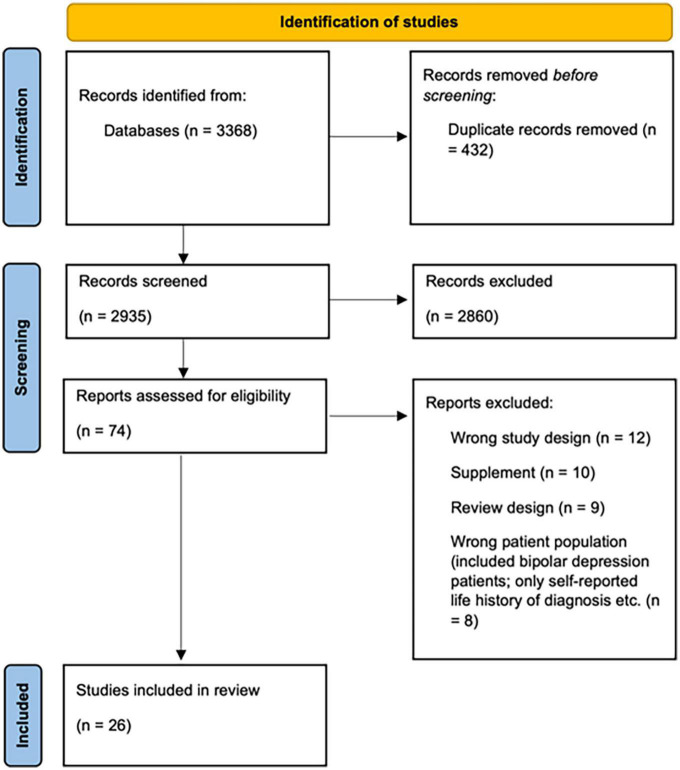
Prisma flow chart shows the process of identification of studies.

Of the 3,368 references retrieved from all the bibliographic databases, we identified 26 eligible studies that met the inclusion criteria. A PRISMA flowchart (see Figure 1) summarizes the search process and presents reasons for exclusion of the studies. After the automatic duplicate elimination, the search consisted of 2,935 studies that were screened against the title and abstract. After the irrelevant articles elimination, the search procedure yielded 74 studies that were assessed for full-text eligibility. In the process of full-text screening, 48 studies were excluded due to the following reasons: 12 did not possess an appropriate study design; 10 were conference abstracts; 9 were reviews; 8 included ineligible patient population; 3 presented at-risk for PPD population only; 3 were duplicates; 3 did not have a between-group design. Of the full-text screening, 26 studies (762 women with PPD and 953 controls in total) met the inclusion criteria and were included in the final study sample. Of these 26 identified studies, 17 were not reviewed previously by Duan et al. (11).

The included studies were of medium to high quality with the lowest score of 6 and the highest score of 12.5. They lacked mostly prospective study design and often did not show coordinates for localization of brain regions in the standard space. On the other hand, the studies generally used standardized methods for PPD diagnosis, they were controlled for the medication status and comorbidities. The studies tended to describe clearly the imaging techniques protocol to be potentially reproducible, provided statistical parameters for observed differences, offered conclusions consistent with results, and discussed their limitations. For full quality data assessment of each study, see the Supplementary material 2.

### Functional magnetic resonance imaging studies

Functional connectivity research provides us new insights how the local and large-scale neural communication and information integration relate to human mental states and behavior via measuring cerebral blood-oxygenation-level (BOLD) dependent changes of neural activity in response to various activities or rest over a time resolution of seconds (16). Recently, there has been a growing body of fMRI research in PPD. We included 16 fMRI studies (10 resting-state, 6 of them task-related) for the final data analysis. Of the 16 studies, we identified 11 that were not part of the review by Duan et al. (11). The list of identified fMRI studies with data extraction for each study is presented in Table 1.

**TABLE 1:** Data extraction of functional magnetic resonance studies.

Lead author	Methods	Paradigm	Sample	Diagnosis and assessment of depressive symptoms	Time after birth	Coordinates	Medication	Comorbidity	Quality assessment score (0-14)
Chase et al. (22)	BOLD resting-state fMRI (whole brain and then ROIs: amygdala subregions)	Resting state	PPD (14) vs. HC (23)	Structured clinical interview (DSM-4) HAM-D > 15	12 weeks	No data	None	None	9.5
Che et al. (23)	BOLD resting-state fMRI (whole brain, exploratory)	Resting state	PPD (16) vs. HC (16)	Structured clinical interview (DSM-5) HAM-D > 20 EPDS > 12	12 months	yes	None	None	12.5
Cheng et al. (25)	BOLD resting-state fMRI (whole brain, exploratory)	Resting state	PPD (45) vs. PPD with anxiety (31) vs. HC (62)	Structured clinical interview (DSM-5 and CCMD-3) EPDS, BAI	within 12 months	No data	None	None (except 1 group had anxiety as comorbidity)	10.5
Cheng et al. (24)	BOLD resting-state fMRI (whole brain, exploratory)	Resting state	PPD (45) vs. PPD with anxiety (31) vs. HC (62)	Structured clinical interview (DSM-5 and CCMD-3) EPDS; BAI	within 12 months	No data	None	None (except 1 group had anxiety as comorbidity)	11.5
Cheng et al. (21)	BOLD resting-state fMRI (whole brain, exploratory)	Resting state	PPD (45) vs. PPD with anxiety (31) vs. HC (62)	Structured clinical interview (DSM-4) EPDS; BAI	within 12 months	yes	None	PPD with anxiety	12
Deligiannidis et al. (18)	BOLD resting-state fMRI (whole brain, exploratory)	Resting state	PPD (23) vs. HC (28)	Structured clinical interview (DSM-4) HAM-D17; HAM-A; EPDS	within 8 weeks	yes	None	None	12.5
Mao et al. (19)	BOLD resting-state fMRI (whole brain, exploratory); Structural MRI	Resting state	PPD (21) vs. HC (23)	Structured clinical interview (DSM-5) HAM-D > 20; EPDS ≥ 12	within 12 months	No data	None	None	11
Schnakenberg et al. (27)	BOLD resting-state fMRI Structural MRI	Resting state	PPD (21) vs. HC (117)	Structured clinical interview, HAM-D > 21; EPDS	6 days	yes	No data	No data	8.5
Wang et al. (17)	BOLD resting-state fMRI	Resting state	PPD (10) vs. HC (11)	Structured clinical interview (DSM-4 and CCMD-3)	within 16 weeks	yes	None	None	10
Zhang et al. (20)	BOLD resting-state fMRI (whole brain, exploratory)	Resting state	PPD (28) vs. HC (25)	Structured clinical interview (DSM-5 and CCMD-3); EPDS ≥ 13	within 1 month	yes	None	None	12
Dudin et al. (34)	BOLD task-related fMRI (ROIs: amygdala)	Emotional task	PPD (32) vs. HC (25)	Structured clinical interview (DSM-4) EPDS; STAI-T	2-5 months	No data	37.5% PPD mothers were taking SSRIs	None	8.5
Ho and Swain (32)	BOLD task-related fMRI (ROIs: amygdala, NAc, dmPFC)	Emotional task	PPD (14) vs. HC (15)	Screening interview (for diagnostic history) BDI > 11	within 4 months	yes	No data	No data	6
Moses-Kolko et al. (31)	BOLD task-related fMRI (ROIs: bilateral dorsomedial PFC, amygdala)	Emotional task	PPD (14) vs. HC (16)	Structured clinical interview (DSM-4) HAM-D ≥ 15; EPDS	4−13 weeks	yes	None	PPD with anxiety disorders	9.5
Silverman et al. (30)	BOLD task-related fMRI	Emotional task	PPD (4) vs. HC (4)	Structured clinical interview (DSM-4) HAM-D34; EPDS > 12	7−8 weeks	yes	None	None	10
Wonch et al. (33)	BOLD task-related fMRI (ROIs: amygdala)	Emotional task	PPD (28) vs. HC (17)	Structured clinical interview (CIDI-5 and DSM-4) EPDS; STAI-T	2−5 months	yes	SSRI (13) vs. no medication (18) in PPD group	No data	9
Moses-Kolko et al. (35)	BOLD task-related fMRI (ROIs: left ventral striatum)	Reward task	PPD (12) vs. HC (12)	Structured clinical interview (DSM-4) HAM-D ≥ 15	within 10 weeks	yes	None	ADHD (*n* = 1), anxiety disorder (*n* = 11), eating disorder (*n* = 2) in PPD	9.5

ADHD, Attentional Deficit Hyperactivity Disorder; BAI, Beck Anxiety Inventory; BOLD, Blood Oxygenation Level Dependent; CCMD, Chinese Classification of Mental Disorders; DSM, Diagnostic and Statistical Manual of Mental Disorders; EPDS, Edinburg Postnatal Depression Scale; fMRI, functional magnetic resonance imaging; HAM-A, Hamilton Anxiety Rating Scale; HAM-D, Hamilton Depression Rating Scale; HC, healthy controls; PFC, prefrontal cortex; PPD, postpartum depression; ROIs, regions of interest; SSRI, Selective Serotonin Reuptake Inhibitors; STAI-T, State-Trait Anxiety Inventory.

#### Resting-state paradigm

In the resting-state paradigm (rs), participants were asked to stay calm and not to think about anything. We have identified 10 studies with the total sample of 361 PPD-diagnosed women and 429 controls that examined fMRI changes in PPD during the resting state. Seven of the studies indicated alterations of the default mode network (DMN) which was mostly hyperactivated in women with PPD. Specifically, there were changes in connectivity and activity in the medial prefrontal cortex (mPFC) (17–21), the posterior cingulate cortex (PCC) (17, 22) and the precuneus (23). Furthermore, regarding the salient network (SN) disruption during rest in PPD, we could affirm differences in the PPD sample in comparison to controls in both major nodes of this network – the insula (19, 24) and the anterior cingulate cortex (ACC) (19, 20, 24). The temporal lobe exhibited repeatedly changes in women with PPD (17–19, 22, 24, 25), mostly suggesting attenuated spontaneous activity. The dorsolateral prefrontal cortex (dlPFC), a cognitive control network hub, showed elevated activity during the rest in PPD (23). Finally, Cheng et al. (21) demonstrated the ventral striatum connectivity disruption in the fronto-striatal neural circuit in women with PPD. We provide more detailed results of each identified rs fMRI study below.

Wang et al. (17) were the first who investigated rs functional connectivity in depressed postpartum women. They detected functional changes in the mPFC, and the PCC in women with PPD in comparison to healthy controls. Furthermore, changes in the temporal lobe observed in women with PPD suggested attenuated spontaneous activity.

Chase et al. (22) focused specifically on the DMN, an anatomically defined brain network mainly comprised of the mPFC, the PCC, the anterior cingulate cortex (ACC), the inferior parietal cortex and the precuneus. The DMN participates in internal modes of cognition, and it is preferentially active when there is no need to be alert toward external stimuli (26). The DMN is rather active when individuals are engaged in internally focused tasks, such as during autobiographic memory processing (26). Chase et al. (22) demonstrated significant disruption in the PCC-right amygdala connectivity in depressed postpartum women compared to healthy controls. Since there is no direct connection between the PCC and the amygdala, they looked for a possible role of the ACC and/or the parahippocampus as the anatomical mediators in the potential polysynaptic pathway between the PCC and the amygdala. The disrupted PCC and right amygdala connectivity correlated with the PCC-parahippocampus gyrus/subiculum connectivity, but not with the PCC-ACC connectivity. Therefore, the parahippocampus rather than the ACC would be possibly involved in this polysynaptic pathway. Authors speculated that the disrupted amygdala-PCC connectivity in women with PPD might reflect reduced ability of orientation of cognition toward the others, especially the infants. Alternatively, the observed PCC-amygdala disruption might be just a basis of the cognitive deficit in PPD in general, not directly connected to the social domain.

Deligiannidis et al. (18) also examined the abnormal activity within the DMN in PPD-diagnosed women. They found that the dmPFC of women with PPD had greater connectivity with the rest of the DMN. Moreover, considering all the postpartum women there was a positive correlation between depression severity and rs functional connectivity within the dmPFC (18).

The role of the DMN in the pathophysiology of PPD was further corroborated by Che et al. (23), who demonstrated increased spontaneous neural activity in the left middle frontal gyrus, the left precuneus, the left inferior parietal lobe, and the left dlPFC in women with PPD. They also showed decreased spontaneous neural activity in the bilateral precentral gyrus, the right inferior occipital gyrus, the right inferior frontal gyrus, and the left cerebellum inferior semilunar lobe. Authors specifically emphasized the possible role of the precuneus (a part of the DMN), which exhibited abnormal regional homogeneity (ReHo) values, and also the role of the dlPFC, which is responsible for executive and cognitive control functioning. Furthermore, an altered function of the superior frontal gyrus, a key component in the process of behavioral responding to emotional stimuli, was also observed in this study (23).

Mao et al. (19) reported changes in the amygdala, the cingulum gyrus, the insula, the hippocampus, the frontal lobe, the parietal lobe, and the occipital lobe in women with PPD as they exhibited altered information flow patterns in these regions compared to non-depressed postpartum controls. The preferred information flow direction between the amygdala and temporal and frontal lobes significantly correlated with depression severity.

Zhang et al. (20) investigated interhemispheric functional connectivity in women with PPD. They found decreased homologous rs functional connectivity in the bilateral dmPFC, the dorsal ACC, and the orbitofrontal cortex (OFC) in PPD. Moreover, the functional connectivity in the dmPFC negatively correlated with depression severity.

In contrast to the previous findings, Schnakenberg et al. (27) found no difference between non-depressed and depressed postpartum women regarding rs functional connectivity. The authors controlled the time course of PPD onset to establish the prognostic value of neuroimaging data, since the women had not been depressed at the time of recruitment but might later become depressed at the time of scanning. Finding no differences, they concluded that rs functional alterations, if present, cannot be used as an early biomarker of PPD.

In a longitudinal perinatal project from Chengdu, China, that aimed to investigate the pathogenetic factors, prognosis, and effective interventions in women with PPD, the fronto-striatal neural circuit with the reward system central hub of the ventral striatum was found to be disrupted in women with PPD (21). The results shed light onto the potential role of hedonism and reinforcement reward disruption in the pathology of PPD. The dmPFC exerts top-down modulation of reward-related firing in the ventral striatum and the striatum simultaneously mediates reward-based motivation in the dmPFC (28). The functional connectivity between these two regions was reduced in women with PPD (21). Furthermore, depressed postpartum women with comorbid anxiety displayed elevated connection between the ventrolateral PFC (vlPFC) and the ventral striatum (21). The activity and connectivity of the lingual gyrus (LG), an area involved in the processing of visual and affective information, were disrupted in PPD women without comorbid anxiety (21).

The same group of Cheng et al. (25) performed yet another analysis of the data from the identical study sample. Women with PPD without comorbid anxiety showed higher Functional Connectivity Strength (FCS) in the parahippocampus, while PPD women with comorbid anxiety showed higher FCS in the left vlPFC (25). The increased vlPFC activity has been previously linked to the anxious phenotype in postpartum mothers, concerning infant caregiving (29). In the study by Cheng et al. (24), depression load was positively correlated with FCS in the left paracentral lobule (pointing once again toward the DMN changes in PPD), and negatively correlated with FCS in the right cerebellum posterior lobe in all postpartum women. More importantly, a subsequent mediation analysis identified perceived social support as the mediator of the FCS influence in the right cerebellum posterior lobe on the PPD symptoms. According to the authors, perceived social support and its underlying neurophysiological correlates might have a major impact in the PPD onset (25), hence could represent a potentially modifiable social target in the PPD prevention.

The authors Cheng et al. (24) followed slightly different cohort of women within the same perinatal project. They observed functional changes in the activity of the subgenual ACC, increased activity, and connectivity between the superior temporal sulcus in women with PPD and comorbid anxiety, and between the ventral anterior insula in PPD without comorbid anxiety. Both groups, depressed women with and without comorbid anxiety, exhibited disrupted functional connectivity between the ACC and the superior temporal gyrus (STG). The deficit in the STG is hypothesized by the authors to be associated with the overall mental representation processing of emotion-related information in PPD and may be related to the feeling of sadness and loss of interest or pleasure (24).

#### Emotional-task paradigm

Emotional task related fMRI research has attracted greater attention than any other type of imaging research in the beginning of the neuroimaging research in PPD. Imaging research of emotion-related tasks mostly focuses on changes in the amygdala activity and connectivity. We identified 5 studies investigating the amygdala (and eventually other related regions), with the total sample of 92 PPD-diagnosed women and 77 controls. The amygdala activity in response to negative emotional stimuli in PPD has been characterized as blunted (30, 31) with the exception of the study by Ho and Swain (32) that, on the contrary, reported elevated activity. While in women with PPD the activity of the amygdala in response to negative emotional stimuli is likely attenuated, it is elevated in response to positive emotional stimuli (33, 34). Furthermore, the dmPFC in PPD-diagnosed women exhibits reduced activity in response to positive emotional stimuli (31) but demonstrates increased functional connectivity in response to negative emotional stimuli (32). Other affected areas in PPD during emotion-related tasks include the OFC (30), the striatum (30), the insula (33), and the NAc (32). We provide more detailed results of each identified emotional-task related fMRI study below.

Silverman et al. (30) were the first to measure neural activity via fMRI during emotional task in women with PPD. They observed attenuated activity in the posterior OFC for negative vs. neutral stimuli, attenuated striatum activity in response to positive word condition with more severe depressive symptoms and reduced amygdala activity in response to negative words in women with PPD.

Moses-Kolko et al. (31) reported earlier reduced dmPFC activity in response to negative emotional faces in PPD. Additionally, depression severity correlated negatively with the left amygdala activity. Moreover, the reliable top-down connectivity from the dmPFC to the left amygdala was also attenuated in women with PPD (31).

Wonch et al. (33) found increased right amygdala activity in response to the positive infant emotional stimuli in women with PPD in comparison to women without PPD. They also reported decreased bilateral amygdala-right insular cortex connectivity in PPD women when viewing their own infant, which was even negatively associated with depression severity.

Ho and Swain (32) examined the changes in the amygdala activity in response to negative emotional stimuli. In their study, depressed postpartum women showed elevated response in the left amygdala under one of the distress conditions and reduced functional coupling between the left amygdala and the nucleus accumbens (NAc) under two of the distress conditions. Additionally, they observed enhanced amygdala functional connectivity with the dmPFC in women with PPD.

An enhanced amygdala response to the unfamiliar infant smiling picture in PPD women in contrast to both non-depressed postpartum women and non-postpartum depressed women was also reported in the study by Dudin et al. (34). The results further support the dissociation of amygdala engagement in respect to positive and negative emotional stimuli in PPD.

#### Reward-task paradigm

There is a lack of studies focusing on the reward-task functional correlates in women with PPD. We identified only one study with such a design (35) where 12 women with PPD exhibited rapider attenuation in the left striatum activity compared to 12 healthy controls (see below).

The motivational function of a response to infant’s needs, which is represented by a reward-driven neural mechanism, appears to be a core tool in the establishment of the mother-infant dyad and it is presumably impaired in PPD (30, 31). A hypothesis of the decreased ventral striatal activity and attenuated dopamine release in PPD in contrast to normal maternal attachment was tested in the study by Moses-Kolko et al. (35) with fMRI scanning during the monetary reward task. The authors reported a normal initial positive BOLD peak in the left ventral striatum in response to a reward followed by significantly faster attenuation back to baseline in women with PPD compared to healthy controls. The ventral striatum plays a major role in reward processing, including the infant-related one, and the attenuated ventral striatal activity in response to pleasant stimuli is considered as a MDD biomarker (36). Moses-Kolko and collaborators also speculated that there might be an eminent, but in their design not tested, role of the PFC in motivational learning dysregulation in PPD.

### Structural magnetic resonance imaging studies

Structural MRI allows us to examine the gray matter (GM) with a measure of specific brain regions called regions of interest (ROI) or with a hypothesis-free approach of voxel-based morphometry (VBM) or surface-based measure, or the white matter (WM) morphometry with the determination of the location, orientation, and anisotropy of the WM tracts (37). We identified 6 structural MRI studies of PPD with the total sample of 178 PPD-diagnosed women and 317 controls. The list of identified structural MRI studies with data extraction for each study is presented in Table 2.

**TABLE 2:** Data extraction of structural magnetic resonance studies.

Lead author	Methods	Paradigm	Sample	Diagnosis and assessment of depressive symptoms	Time after birth	Coordinates	Medication	Comorbidity	Quality assessment score (0−14)
Cheng et al. (41)	Structural MRI	Voxel-based morphometry	PPD (86) vs. HC (74)	Structured clinical interview (DSM-5 and CCMD-3); EPDS	3 to 5 months	yes	None	None	12.5
Li et al. (40)	Structural MRI (whole brain)	Structural covariance network	PPD (21) vs. HC (18)	Structured clinical interview (DSM-5) HAM-D > 20; EPDS ≥ 12	within 12 months	yes	None	None	11
Li et al. (39)	Structural MRI (whole brain)	Surface-based morphometry	PPD (21) vs. HC (18)	Structured clinical interview (DSM-5) HAM-D > 20; EPDS ≥ 12	within 12 months	yes	None	None	12
Mao et al. (19)	Structural MRI (whole brain)	Preferred information flow	PPD (21) vs. HC (23)	Structured clinical interview (DSM-5) HAM-D > 20; EPDS ≥ 12	within 12 months	No data	None	None	11
Sasaki et al. (38)	Diffusion kurtosis imaging (ROIs: caudate nucleus, putamen, globus pallidus, thalamus)	White matter [mean kurtosis (MK), FA, and mean diffusivity (MD)]	PPD (8) vs. HC (67)	Structured clinical interview (DSM-5); EPDS	1 month	No data	No data	None	9
Schnakenberg et al. (27)	Structural MRI	Voxel-based morphometry	PPD (21) vs. HC (117)	Structured clinical interview; HAM-D21; EPDS	6 days	yes	No data	No data	8.5

CCMD, Chinese Classification of Mental Disorders; DSM, Diagnostic and Statistical Manual of Mental Disorders; EPDS, Edinburg Postnatal Depression Scale; HAM-D, Hamilton Depression Rating Scale; HC, healthy controls; MRI, magnetic resonance imaging; PPD, postpartum depression; ROIs, regions of interest.

The results of structural MRI studies of PPD are inconsistent. One structural study detected the changes of the white matter structure in various brain regions (e.g., parietal, occipital, temporal, and frontal cortices) (38), while other studies (39) suggested structural connectivity alterations in the DMN, the CCN or the visual system, the surface-area increase in the frontal lobe and temporal lobe (40) or increase of the GM volume in the dlPFC and the insula (41). We provide more detailed results of each identified structural MRI study below.

It was not until 2020, when Sasaki et al. (38) used for the first time an advanced form of diffusion tensor imaging (DTI), diffusion kurtosis imaging (DKI), to assess structural signatures of PPD. Their principal findings were a significant decrease of a fractional anisotropy (FA) value within the superior longitudinal fasciculus, the corticospinal tract, and the thalamus; a significant increase of a mean diffusivity (MD) value within the white the matter of the temporo-parietal regions, the superior longitudinal fasciculus, the corticospinal tract, the cingulum, and the putamen. Overall, they did not find any difference in mean kurtosis (MK) value in women with PPD compared to healthy controls.

Li et al. (39) investigated brain structural covariance networks that enables to characterize the topological properties of the brain organization presumably associated with the PPD structural pathophysiology. There was a PPD-related hub in the inferior parietal lobule (IPL). Additionally, women with PPD had decreased connections in the CCN and visual system (VIS) and increased connections in the DMN. The same group of authors performed yet another type of analysis in the same sample of women (40). They observed increased surface area in the left superior frontal gyrus, caudal middle frontal gyrus, middle temporal gyrus, and insula. Furthermore, cortical abnormalities in the left insula and superior parietal lobule were correlated with the depression severity (40). Considering these results, the authors concluded that PPD manifests itself as a system-level disorder possibly influencing various brain networks.

Cheng et al. (41) reported increased GM volume in the left dlPFC in women with PPD compared to healthy controls. The increased GM volume in the insula was positively correlated with the depression severity. Furthermore, mediation analyses revealed that prolactin level mediates the association between GM volume in the right insula and depression severity in PPD. The mediating role of prolactin level presents an important finding as sexual hormonal fluctuation is considered a significant risk factor for the PPD onset.

In contrast to the studies that found structural correlates of PPD, Schnakenberg et al. (27) failed to detect any difference between depressed and healthy mothers.

### Metabolite studies

Proton magnetic resonance spectroscopy (1H-MRS) and positron emission tomography (PET) provide biochemical evaluation of specific brain regions, enabling assessment of the viability, neurotransmission, energy metabolism, damage processes and cellular renewal of the underlying neuronal tissue. Hence, these imaging techniques are great tools to examine metabolic changes in PPD and psychiatric disorders in general (42, 43). We identified 7 studies measuring metabolite changes in PPD with the total sample of 119 PPD-diagnosed women and 118 controls. The list of all metabolic studies with data extraction for each study is presented in Table 3.

**TABLE 3:** Data extraction of metabolic studies.

Lead author	Methods	Paradigm	Sample	Diagnosis and assessment of depressive symptoms	Time after birth	Coordinates	Medication	Comorbidity	Quality assessment score (0−14)
Deligiannidis et al. (18)	MRS (ROIs: pregenual ACC and occipital cortices)	Cortical GABA concentration	PPD (23) vs. HC (28)	Structured clinical interview (DSM-4) HAM-D17; HAM-A; EPDS	within 8 weeks	yes	None	None	12.5
de Rezende et al. (51)	MRS (ROIs: ACG)	Various metabolite levels	PPD (20) vs. HC (19)	Structured clinical interview (DSM-4) EPDS	within 6 months	No data	None	PPD with anxiety disorders	9.5
Epperson et al. (47)	MRS (ROIs: occipital cortex)	GABA concentration	PPD (9) vs. HC (14)	Structured clinical interview; HAM-D ≥ 18	within 6 months	No data	None	None	8
McEwen et al. (45)	MRS (ROIs: mPFC)	Metabolites of glutamate	PPD (12) vs. HC (12)	Structured clinical interview (DSM-4) EPDS; BDI	3 weeks to 3 months	No data	None	None	8
Moses-Kolko et al. (48)	PET (ROIs: striatum, caudate, putamen)	D2/3 receptor binding potential	PPD (13) vs. HC (13)	Structured clinical interview (DSM-4) HAM-D17≥14 HAM-D25≥18	within 16 weeks	No data	None	No data	7.5
Moses-Kolko et al. (44)	PET (ROIs: mesiotemporal cortex, lateral OFC, subgenus ACC)	Serotonin 5HT1A receptor binding potential	PPD (9) vs. HC (7)	Structured clinical interview (DSM-4) HAM-D25 ≥ 14	within 16 weeks	No data	None	Past substance use disorders (*n* = 5), anxiety disorders (*n* = 7) in PPD	7
Rosa et al. (46)	MRS (ROIs: ACC, dlPFC)	Glutamate + glutamine metabolites	PPD (33) vs. HC (25)	Structured clinical interview (DSM-4) EPDS; HAM-D17; BAI	up to 6 months	No data	None	mild anxiety disorders – 10 (HC), 17 (PPD)	10

ACC, anterior cingulate cortex; ACG, anterior cingulate gyrus; BAI, Beck Anxiety Inventory; BDI, Beck Depression Inventory; D2/3 receptor, dopamine receptors; dlPFC, dorsolateral prefrontal cortex; DSM, Diagnostic and Statistical Manual of Mental Disorders; EPDS, Edinburg Postnatal Depression Scale; GABA, gamma-aminobutyric acid; HAM-A, Hamilton Anxiety Rating Scale; HAM-D, Hamilton Depression Rating Scale; HC, healthy controls; mPFC, medial prefrontal cortex; MRS, magnetic resonance spectroscopy; OFC, orbitofrontal cortex; PET, positron emission tomography; PPD, postpartum depression; ROIs, regions of interest.

Some cortical regions (the ACC and the OCC, etc.) showed reduced serotonin-1A (5HT1A) receptor binding potential (44), while others (vmPFC and dlPFC, etc.) displayed changes in glutamate levels in women with PPD, increase and decrease, respectively (45, 46). There was no dysregulation of the GABA (47) or dopamine (48) systems observed in PPD women. We provide the results of each identified metabolite study in detail below.

Epperson et al. (47) examined a potential alteration in the gamma-aminobutyric acid (GABA) concentrations in the occipital cortex of women with PPD using 1H-MRS. The neural inhibitory function of GABA is essential for synchronized activity in the CNS and the changes in GABA levels and consequently the inhibitory dysregulation have been reported previously in various psychiatric conditions (e.g., autism spectrum disorders, MDD) (49). In the study by Epperson et al. (47), both groups of postpartum women (depressed and non-depressed) had lower GABA concentrations in the occipital cortex compared to non-postpartum women (47). There was, however, no difference in the GABA levels between the two postpartum groups.

Serotonin system and its role in the pathophysiology of MDD and mood regulation has been intensively studied; thus, serotonin pathology is also of interest in PPD research. Moses-Kolko et al. (44) measured brain serotonin-1A (5HT1A) receptor binding potential with PET in a small study with postpartum women. The authors found out that postsynaptic 5HT1A receptor binding was reduced by 20−28% in PPD women relative to healthy postpartum controls, especially in regions of the ACC, the mesiotemporal and the lateral orbitofrontal cortices.

McEwen et al. (45) reported higher glutamate levels in the mPFC in women with PPD in comparison to healthy controls. Interestingly, an earlier study with MDD patients (both males and females) found reduced glutamate levels in the cingulum and the higher levels predicted therapeutic response to electroconvulsive therapy (50).

Moses-Kolko et al. (48) assumed the dopaminergic reward system to be disrupted in PPD. However, even though unipolar depression in general was associated with lower D2/3 receptor binding in the striatum, a region implicated in the reward system, no difference from women with no diagnosis was observed in PPD patients.

Rosa et al. (46) additionally reported reduction in the levels of “glutamate complex” (Glx, i.e., glutamate + glutamine) and “NAA complex” (N-acetylaspartate + N-acetylaspartylglutamate) in women with PPD in the left dlPFC.

Steele et al. (51) targeted the ACC as a region of interest to assess various metabolites via 1H-MRS, but failed to find any significant difference between women with and without PPD. However, they observed that decreased hypothalamic-pituitary-adrenal (HPA) axis responsiveness in PPD women was associated with lower concentrations of Glx metabolites in the anterior cingulate gyrus (ACG).

Deligiannidis et al. (18) did not confirm reduced cortical GABA concentrations in women with PPD as hypothesized, since they are naturally raised postnatally. However, regardless of mood, the ACC and occipital cortex (OCC), GABA + /Creatine (Cr) concentrations were associated with intrinsic connectivity of the dmPFC in women with PPD.

### Summary of the review results

**FIGURE 2:**
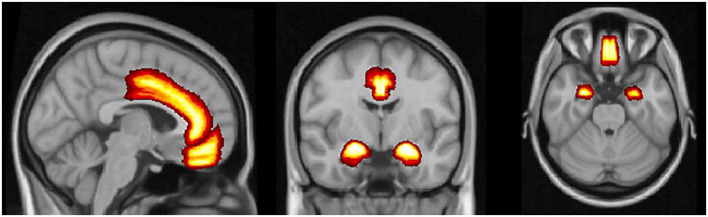
The medial prefrontal cortex, the anterior cingulate cortex, and amygdala. Brain regions with the most consistent neuroimaging and metabolic findings that differentiate women with postpartum depression from healthy controls.

In our review, we identified 3 brain regions that showed consistent differences in women with PPD from healthy controls, based on fMRI results. The most significant differences in fMRI studies were detected in the mPFC, a DMN hub (5 of 10 included studies showed significant difference during the state of rest and 2 of 5 studies during emotional tasks), and the ACC, a SN hub (3 of 10 included studies showed significant difference during the state of rest); 2 studies additionally showed difference in metabolic function of these brain regions in the PPD group. Furthermore, the amygdala showed difference in all 5 identified studies that employed emotional task design. The structural and metabolite findings were not overly consistent in our review; it can be attributed to the fact that authors preferentially analyzed different regions of interest. Figure 2 displays the brain regions with the most consistent findings of the reviewed studies.

## Discussion

In the current systematic review, we have collected studies covering the topic of neural signatures (i.e., structural, functional, and metabolic correlates) in postpartum depression (PPD) with a perinatal onset up to 12 months after the delivery and summarized their principal findings. Throughout the years, major depressive disorder (MDD) has been characterized as a complex disorder with underlying changes preferentially detectable in the default mode network (DMN), the salience network (SN), and the cognitive control network (CCN) (52). Therefore, in our review we used these networks to frame the discussion of the PPD signatures. Moreover, we compared the imaging findings of PPD with MDD. Such a comparison can give us a better understanding of the unique and/or shared features of PPD and MDD which could help to devise effective treatment and preventive strategies.

### Default mode network

The DMN is a fMRI-measurable network of a highly interconnected set of brain hubs, mainly active during the rest and mostly suppressed during the tasks which demand focus of attention toward external stimuli (26).

Various studies have concurred that the hyperactivity of the DMN represents the most common functional network feature of MDD (53–56). This finding is further supported by the restored functional activity following antidepressant treatment in patients with MDD (57). In our systematic review, we also confirmed the DMN hyperactivity as the most frequently reported functional feature of PPD. The best examined nodes are the anterior cingulate cortex (ACC), the medial prefrontal cortex (mPFC), the posterior cingulate cortex (PCC), the orbitofrontal cortex (OFC), and the precuneus. The hyperactivity within the DMN seems to be a shared neural correlate for both PPD and MDD in general, not related to the peripartum period. Furthermore, Li et al. (40) found decreased cortical thickness in the right inferior parietal lobule (IPL), another DMN-related structure, in women with PPD. The cortical changes in the IPL were also observed in MDD (58). A small PET study showed reduced postsynaptic 5HT1A receptor binding potential in the ACC and the OFC in women with PPD (44). Maladaptation to motherhood with the lack of ability to be socially sensitive and responsible may stem from social cognition dysregulation as a consequence of the amygdala-PCC connectivity disruption in women with PPD (22).

The dorsomedial prefrontal cortex (dmPFC) dysfunction has been previously associated with MDD (56, 59). In line with these results, disturbances of the dmPFC have been also reported in the available PPD literature. The dmPFC was implicated in hyperactivity within the DMN, particularly the precuneus (18), and exhibited decreased bilateral functional connectivity (20). Moreover, the reduced activity of the dmPFC in response to negative emotional stimuli might represent diminished awareness toward the infant in the processing of emotions in PPD, since the disrupted dmPFC-amygdala connectivity results in emotional dysregulation and reappraisal difficulties of negative valence (31). Reduced connectivity between the amygdala and the mPFC was also evidenced by the decreased white matter integrity between these two regions in PPD (60). However, Ho and Swain (32) reported on the contrary increased functional connectivity between the amygdala and the dmPFC, in response to negative, not positive, emotional stimulus. Cheng et al. (21) additionally suggested functional implication of the dmPFC in the disruption of the reward system in PPD. Interestingly, in contrast to MDD studies (61, 62), McEwan et al. (45) reported higher Glu levels in the mPFC in women with PPD. Abnormally elevated resting-state (rs) activity of the mPFC might be involved in the depression-related rumination (56, 63). On the other hand, gentle touch and bonding activate the anterior mPFC in infancy (64). Since its hyperactivity has been associated with more severe depressive symptoms (65), it seems that some of the brain-related alterations could be positive for caregiving, allowing affected women to allocate more resources to the bonding with the infants. An alternative explanation is that it is just a compensation of their lower ability to express and regulate emotions. Lastly, the increased deactivation of the DMN from rest to task-related activity in depressed postpartum women might represent the disruption of the ability to shift from internally to externally focused attention for the infant (66).

### Salience network

The Salience network (SN) refers to a network with cortical central hubs of the ACC and the ventral anterior insular cortex and additional nodes in the amygdala, hypothalamus, ventral striatum, thalamus, and specific brainstem nuclei. The SN plays a key role in integrating and transferring information between different functional modules. Additionally, the SN selects to which stimuli is our attention assigned and consequently coordinates the behavioral response to these “salient” stimuli (67).

Cortical structures such as the ACC work as a regulator of the limbic structures and as so they co-determine the emotional stimuli processing. Some depressive characteristics (e.g., negative interpersonal bias) might be explained by a breakdown in this cortico-limbic circuit (68). The specific function of the ACC is a modulation of behavior in response to emotions. Moreover, the ACC is included in the reward, fear, and the stress system (69, 70). In our review, the ACC exhibited several functional abnormalities in women with PPD, analogously to MDD studies was associated with the hyperactivity (24, 53, 68, 71). Furthermore, the ACC manifested decreased connectivity with the dmPFC (20), and increased connectivity with the superior temporal sulcus and the ventral anterior insula (24). The ACC-insula connectivity was similarly disrupted in MDD patients (72, 73). Apart from that, the ACC showed reduced serotonin 1A receptor binding (44), but no changes in Glu and other metabolite levels (46, 51), or GABA concentration (18) in women with PPD.

The insula is a key component of affective, memory and saliency processing. In our review, we found the disruption in the insula activity and structure to be a common correlate of PPD, which corresponds to the MDD studies (74–76). The exact mechanism how the insula determines emotional dysregulation in PPD is difficult to clarify, since the studies showed its decreased connectivity to the amygdala and the frontal cortices (19, 77), but increased connectivity to the ACC (24). Due to the insula role in a regulation of emotions, its abnormal connectivity might be responsible for affective symptoms such as somatic complaints and negative bias interpreting interpersonal feedback in PPD (19). Moreover, the insula of PPD women in our review also had increased gray matter (GM) volume (41) and increased cortical surface area (40), suggesting it plays an important role in the pathogenesis of PPD.

### Cognitive control network

The Cognitive control network (CCN) is a brain network comprising of the dorsolateral prefrontal cortex (dlPFC), the dorsal anterior cingulate cortex (dACC) and the parietal cortex. The CCN is preferentially active during tasks as it employs executive functions, such as attention, flexibility, planning, or working memory, to guide an appropriate behavior in order to achieve a specific goal (78).

Disruption of the ability to control the executive inhibition and the elevated anxiety anticipation is mostly attributed to the left dlPFC hypoactivity in MDD patients (56, 79), which is restored after antidepressant treatment (80). In our review, we identified one study (23) in which PPD women showed increased activity in the left dlPFC during rs, in contrast to the hypoactivity observed in MDD (79). This finding suggests that PPD could be a specific subtype of depressive disorder. However, the finding still needs to be replicated in order to confirm or disprove this hypothesis. Based on their results, Harvey et al. (81) suggested that MDD patients need greater dlPFC activity to maintain a similar level of the CCN related performance. Interestingly, the PPD women exhibited the hyperactivity during rest, not during the executive task-related state as in the study by Harvey et al. (81). More studies, during both task and resting-state, need to be done in order to understand the dlPFC functional changes in PPD. In our review, we also observed that the dlPFC of the PPD women showed increased regional GM volume (41), lower Glu metabolism and decrease in the levels of “NAA complex” (N-acetylaspartate + N-acetylaspartylglutamate) (46), similarly to the patients in MDD studies (61, 82, 83).

### Corticolimbic system

Emotional dysregulation is considered to be one of the core features of depression. Understanding of the underlying neural basis of the impairment of emotional processing in depression could help us to uncover some of the leading causes/consequences of this debilitating disorder. Bottom-up emotional regulation is mostly influenced by the amygdala, the insula, the ventral striatum, the anterior cingulate gyrus (ACG), the ventromedial prefrontal cortex (vmPFC), and the OFC. These anatomical structures are thought to play a pivotal role in recognizing emotionally salient stimuli and they subsequently cooperate to generate the overall emotional state. Top-down emotional regulation allows to voluntary control emotions, preferentially through the activity of the hippocampus, the ACC, and the PFC. These two systems operate parallel and simultaneously (84).

The amygdala as an important subcortical structure in generating emotions is of major interest in MDD research. Published studies suggested that in patients with MDD the amygdala is hyperactivated in response to negative stimuli, and the activation is extended and persists even after the negative stimuli is no longer present (85–87). The amygdala hyperactivity is assumed to be a main functional component of the negative emotional processing in MDD; thus, we expected to detect similar disruption in women with PPD. However, the data suggest a different direction of the amygdala response to negative emotional stimuli in PPD, an attenuated activity in PPD (30, 31), one study yielded non-consistent results (32). Furthermore, Barrett et al. (88) showed that poorer quality of maternal experiences was related to the attenuated activity in the amygdala in response to emotional stimuli of infants’ faces. In response to positive emotional stimuli, there is an increased activity in the amygdala in PPD (34, 77) in contrast to attenuated activity in MDD (89).

Therefore, these results might be considered as a specific neural correlate distinguishing PPD from general non-peripartum MDD. However, additional studies with a design incorporating various emotional stimuli are needed, since not all the PPD studies provided consistent results (see ref. 32). The amygdala has also an important function in threat processing, while the nucleus accumbens (NAc) in reward processing (32). The functional reward processing is needed for sensitive parenting and the diminished functional connectivity between the amygdala and the NAc in PPD when hearing own baby’s cry could represent the underlying neural mechanism of difficulties to integrate baby-related negative stimulus into the reward processing (32).

The ventral striatum, which is predominantly involved in positive information processing, is less sensitive in depressed patients (90). Similarly to MDD (55), women with PPD exhibited disruption in the ventral striatum activity (21, 30). In mothers, a pleasant stimulus, such as an infant smiling, activates the ventral striatum (91); therefore, attenuated response of the striatum might result in caregiving difficulties. Indeed, more severe symptoms of PPD were associated with lower ventral and dorsal striatal activity in response to positive emotional stimuli in the study by Morgan et al. (92). Such lower striatal activity indicates the existence of blunted reward functioning in PPD women. In addition, Moses-Kolko et al. (48) failed to detect lower D2/3 receptor binding in the striatum in PPD women when they were examined separately from women with non-peripartum unipolar depression, which might be attributed to the small sample size (48).

The ventrolateral prefrontal cortex (vlPFC) works as a value assigner and the negative attention bias is thought to stem from reduced activity in this brain region (93). The vlPFC activity was elevated in women with PPD, but only in those with the comorbid anxiety, when compared to PPD-diagnosed women without comorbid anxiety or non-depressed women (25). The elevated vlPFC activity could be, along with the straightened connection between the vlPFC and the ventral striatum (21), recognized as a paradox compensatory mechanism in the in the regulation of depression-related anxiety.

The OFC serves as an emotional inhibitory regulator and influences emotional decision making (94, 95). The attenuated posterior OFC activity is a feature of MDD (96); thus, not surprisingly, women with PPD also displayed its attenuated activity in response to negative emotional stimuli (30) and reduced postsynaptic 5HT1A receptor binding potential (44). Moreover, blunted activity of the OFC was reported in PPD women in response to their own infant’s joy face (97).

### Temporal lobe

Temporal lobe disruption (i.e., alteration in the activity of the hippocampus, the parahippocampal gyrus, the amygdala, and the caudate nucleus) has been previously reported in MDD patients (98, 99). Similarly, our review confirmed impairment in these regions in PPD, as evidenced by the findings from numerous studies (17–19, 22, 24).

### Comparison to the previous comprehensive review

Due to the fact that the majority of the included studies (17/26) were not covered by the previous comprehensive review of the PPD neural correlates Duan et al. (11), our current updated systematic review can further expand our knowledge of the PPD pathogenesis. Since 2017, many high-quality studies have been published on the topic, most notably research using the structural MRI and rs fMRI paradigm. Duan et al. (11) primarily synthetized results from the emotional task-related fMRI studies and therefore emphasized the importance of the amygdala and other limbic structures dysregulation in PPD, while the newly included studies highlighted the changes in the DMN. This can be attributed to the fact that the majority of newly included studies were performed during resting-state when the activity of the DMN is predominating over the activity of other networks (18, 20, 21, 23, 24). Moreover, the recent structural studies were also focused on the DMN structures and were able to corroborate their abnormalities in women with PPD (39, 40). Other currently reviewed studies additionally identified PPD structural correlates in the SN, particularly in the insular cortex, as women with PPD showed increased regional GM volume and increased surface area (40, 41). In the review by Duan et al. (11) the authors were not able to identify a single structural study covering PPD, thereby our structural findings, even though lacking outcome consistencies, present a valuable contribution to the PPD imaging research.

It is also important to mention that in our review sample, we included studies with a more conservative approach in PPD diagnostics. Specifically, for women to be included in the PPD group they had to undergone a formal diagnostics interview. A self-reference or scoring above the threshold in the EPDS or other screening scales alone was not sufficient for the assignment to the PPD-diagnosed group, in contrast to the review by Duan et al. (11). Because of that, and also due to the fact that Duan et al. (11) not only focused on PPD, but included also antenatal depression, the study samples overlap only partially. Thus, while our review comprises the total number of 762 women with PPD and 953 healthy controls, only 117 of PPD-diagnosed women and 118 healthy controls from this sample have been previously included in the study by Duan and collective. Finally, contrary to the review by Duan et al. (11), our review offers a comparison between the PPD and MDD neural correlates in the discussion.

### Limitations

Even though we used very conservative approach regarding the inclusion and exclusion criteria, the reviewed studies are still too heterogeneous in terms of their characteristics to draw unequivocal conclusions. First, the imaging parameters, such as scanner strength, slice thickness, or imaging sequences, varied across the studies. Second, there was a great variability in the study designs. For instance, the severity of PPD differed among the studies, included were women with mild, moderate, or severe depression. The duration of disorder also varied, some studies included first-episode depression patients only, while others did not distinguish between chronic and acute disorder state.

There was also a great variability in methods used to determine the diagnosis of PPD. For consistency, we excluded studies in which the diagnosis was based on non-validated diagnostic methods. Studies included in the review mostly used structured clinical interview, others relied on standardized rating scales, such as the Hamilton Depression Rating Scale (HAM-D), the Edinburg Postnatal Depression Scale (EPDS) or the Beck’s Depression Inventory (BDI), some studies applied both approaches. Since there is still not a generally accepted agreement on the time of onset of depression to be considered PPD, the reported onset ranged from the antenatal period through the first 4 weeks after the delivery up to one year after the childbirth. This fact affects the results and impedes their generalizability. To specify this methodological discrepancy in our review, Schnakenberg et al. (27) obtained the imaging data from women only 6 days after delivery, in order to explore whether fMRI correlates might represent the early PPD biomarkers. However, the early time of the testing might be the reason why they failed to find any difference between women with PPD and healthy controls (27), since the studies that included women up to 12 months after childbirth (e.g., 19, 23, 25) were more likely to find the expected differences.

Another important factor is the medication status as the antidepressant treatment influences functional changes and structural integrity and may thus affect the results (100). Among the reviewed studies, drug-free status had predominantly samples with first-episode patients. Due to the ubiquitous presence of anxiety symptoms in PPD women, it was not possible to exclude all psychiatric comorbidities. Since PPD with anxiety showed different functional phenotypes from PPD without anxiety in the recent fMRI studies (21, 24, 25), this limitation should be taken into consideration. Finally, not only imaging- or design-related factors, but also the statistical approaches varied across the studies, thus it is difficult to synthesize and interpret the results.

It is noteworthy that several studies, namely by Cheng et al. (21, 24, 25), and Li et al. (39, 40) respectively, were conducted by the same group of authors within their longitudinal perinatal projects, using overlapping study samples, but different statistical approaches. For instance, Cheng et al. (21) performed the analysis of Functional Connectivity Strength (FCS) in the first study, the analysis of Functional Connectivity Density (FCD) in the second (25), and the dynamic and static Functional Connectivity analysis in the third study (24). Therefore, the overall interpretation might be influenced by using the same study sample and, at the same, a different data-driven technique. Subsequently, the comprehensive analysis in each of the studies identified many shared (e.g., prefrontal cortex and temporal lobe), but also unique (e.g., lingual gyrus, striatum, and insula) changes in PPD diagnosed women.

Another limitation of numerous included studies, especially those with MRI, was a small study sample. For example, Silver et al. (30) obtained their results from just 4 women with PPD and 4 healthy controls (30). Nevertheless, their preliminary findings, reduced amygdala activity in response to negative words in women with PPD, was later supported by another more credible study with a larger study sample (14 PPD women vs. 16 healthy controls) (31). Additional research is still needed to obtain unequivocal evidence. In summary, the interpretation of our findings (and imaging results in general), which were mostly obtained within the cross-sectional study design, is complicated and prevents from identification of causal relationships. Thus, the summary of the reviewed results does not allow us to determine whether the reported PPD abnormalities represent risk factors or whether the changes emerge during the course of PPD as a result of other underlying pathogenetic processes.

### Future directions

Based on the methodology discrepancies of the PPD studies identified via our search, we propose several recommendations for future research. Investigators should strive to recruit larger, more homogenous samples which will eventually help to eliminate the impact of the clinical confounding factors. First, the clinical group should be defined according to the current versions of diagnostic manuals. All comorbidities, except for the anxiety disorders, should be excluded since they can significantly interfere with the results. Moreover, medication status, the type, and the dose of psychotropic agents, needs to be accounted for.

Whole brain analysis with a free estimation of the depression-related regions always offers less biased results and increased consistency which, in contrast to the region-focused analysis, allows to compare the results directly. Therefore, we suggest applying preferentially this type of analysis. Since the design of our review does not make possible to evaluate the differences between MDD and PPD directly, we suggest employing the between subject design with separate PPD and MDD groups. Such study design could help us to detect better the small, but important nuances between PPD, MDD, and other psychiatric disorders. Even though the number and variety of discrepancies of the findings do not allow us to use the imaging techniques directly in the clinical practice yet, they are important for our better understanding of the nature of PPD. Greater utilization of these techniques in research can, with the help of computer engineering, become in the future a useful clinical tool for PPD diagnostics and prediction of treatment outcome.

## Conclusion

Women with postpartum depression exhibit functional and structural abnormalities preferentially in the default mode network. Furthermore, postpartum depression is associated with structural and functional changes in the salient network. These findings correspond fully to the neuroimaging correlates of major depressive disorder. Additionally, women with postpartum depression manifest attenuated amygdala activity in response to the negative emotional stimuli and increased activity in response to the positive stimuli, which is in opposite direction from the findings in major depressive disorder.

## Data availability statement

The original contributions presented in this study are included in the article/Supplementary material, further inquiries can be directed to the corresponding author/s.

## Author contributions

AH participated on the methodology of the review, search of included studies, and drafting the manuscript of the review. HN participated on the search review process. PM reviewed the draft manuscript and approved the final version of manuscript. AS contributed to the methodology of review, reviewed the draft manuscript, and approved the final manuscript. All authors contributed to the article and approved the submitted version.

## Footnotes

1
www.covidence.org
