# Supplementary material for: Structural, functional, and metabolic signatures of postpartum depression: A systematic review
Source: Front Psychiatry. 2022 Nov 16;13:1044995. doi: 10.3389/fpsyt.2022.1044995 (PMC9709336; doi:10.3389/fpsyt.2022.1044995)
Supplement: Supplementary file 2 [file Table_2.DOCX]

Quality Assessment Checklist

For the assessment of risk of bias, we used quality assessment checklist adapted onto neuroimaging studies evaluation specifically designed by us for the purpose of current review. Two authors independently assessed the quality of studies and agreement between them was required in order to decide the final score. The studies were assessed in 14 items (see supplementary material 1) with the overall score possibly ranging from 0 (very low quality) to14 (very high quality).

**Category 1: Subjects**

1. Subjects evaluated prospectively.

0 Subjects evaluated one time.

0.5 Subjects evaluated two times.

1 Subjects evaluated more than two times.

2. Patients diagnosed with a structured clinical interview.

0 Patients diagnosed without a structured clinical interview.

0.5 Patients diagnosed with a structured clinical interview.

1 Patients diagnosed with a structured clinical interview and also a standardized method for the assessment of depressive symptomatology.

3. Nonpatients without psychiatric history diagnosis and medication-free.

0 No information about nonpatients’ psychiatric history and medication status.

0.5 Nonpatients have either psychiatric history or are medicated.

1 Nonpatients without psychiatric history and medication-free.

4. Demographic data reported.

0 Demographic data not reported or three or less demographic variables presented.

0.5 More than three and less than five demographic variables presented.

1 At least five demographic variables presented.

5. Medication-free patients.

0 Medicated patients or medication status not reported.

0.5 -

1 Medication-free patients.

6. First-episode depression diagnosis with a perinatal onset (pregnancy-12 months postpartum).

0 Time of depression onset not reported.

0.5 Perinatal depression onset with a history of depression.

1 First-episode diagnosis with a perinatal onset (pregnancy-12 months postpartum).

7. Important confounds (e.g., illness duration, medication status, comorbidity, illness severity, number of previous pregnancies) controlled either by stratification or statistically.

0 None of the important confounds controlled.

0.5 At least one important confound controlled.

1 More than one important confound controlled.

8. Sample size per group > 10.

0 Sample size per group < 10

0.5 -

1 Sample size per group > 10

**Category 2: Methods for image acquisition and analysis**

9. Whole brain analysis automated with no apriori regional selection.

0 Yes

1 No

10. Coordinates reported in a standard space.

0 Yes

1 No

11. Imaging technique clearly enough described to be reproduced.

0 Yes

1 No

12. Measurements clearly enough described to be reproduced.

0 Yes

1 No

**Category 3: Results and Conclusions**

13. Statistical parameters provided for significant and important non-significant differences.

0 Yes

1 No

14. Conclusions consistent with results; limitations discussed.

0 Yes

1 No

Score (0 / 0.5 / 1):
