# Supplementary material for: Structural, functional, and metabolic signatures of postpartum depression: A systematic review
Source: Front Psychiatry. 2022 Nov 16;13:1044995. doi: 10.3389/fpsyt.2022.1044995 (PMC9709336; doi:10.3389/fpsyt.2022.1044995)
Supplement: Supplementary file 3 [file Table_3.DOCX]

Quality Assessment Data

The quality of studies was assessed in 14 items (see supplementary material 1) with the overall score possibly ranging from 0 (very low quality) to14 (very high quality). The included studies offered medium to high quality with the lowest score of 6 and highest score of 12.5. The included studies mostly lacked prospective study design and often did not show coordinates for localization of brain regions in the standard space. On the other hand, the studies generally used standardized methods for postpartum depression diagnostics, controlled for the medication status and possible comorbidities. The studies tended to clearly describe the imaging techniques protocol to be potentially reproduced, provided statistical parameters for observed differences, offered conclusions consistent with results, and discussed limitations. Here we present data of full quality assessment in respect to each study.

| Study | Q 1 | Q 2 | Q 3 | Q 4 | Q 5 | Q 6 | Q 7 | Q 8 | Q 9 | Q 10 | Q 11 | Q 12 | Q 13 | Q 14 | Score |
| --- | --- | --- | --- | --- | --- | --- | --- | --- | --- | --- | --- | --- | --- | --- | --- |
| Wang 2011 | 0 | 1 | 1 | 0 | 1 | 1 | 0 | 0 | 1 | 1 | 1 | 1 | 1 | 1 | 10 |
| Cheng 2021 | 0 | 1 | 1 | 0 | 1 | 1 | 1 | 1 | 1 | 1 | 1 | 1 | 1 | 1 | 12 |
| Cheng 2022 | 0 | 1 | 0.5 | 0 | 1 | 1 | 1 | 1 | 1 | 0 | 1 | 1 | 1 | 1 | 10.5 |
| Cheng 2022 | 0 | 1 | 1 | 0 | 1 | 1 | 0.5 | 1 | 1 | 1 | 1 | 1 | 1 | 1 | 11.5 |
| Moses-Kolko 2008 | 0 | 1 | 1 | 0 | 1 | 0 | 0 | 0 | 0 | 0 | 1 | 1 | 1 | 1 | 7 |
| Che 2020 | 0 | 1 | 1 | 0.5 | 1 | 1 | 1 | 1 | 1 | 1 | 1 | 1 | 1 | 1 | 12.5 |
| Moses-Kolko 2010 | 0 | 1 | 1 | 0.5 | 1 | 0 | 0 | 1 | 0 | 1 | 1 | 1 | 1 | 1 | 9,5 |
| Chase 2014 | 0 | 1 | 1 | 0.5 | 1 | 0 | 0 | 1 | 1 | 0 | 1 | 1 | 1 | 1 | 9,5 |
| Deligiannidis 2019 | 1 | 1 | 1 | 1 | 1 | 0.5 | 0 | 1 | 1 | 1 | 1 | 1 | 1 | 1 | 12.5 |
| Epperson 2006 | 0 | 1 | 1 | 0.5 | 1 | 0.5 | 0 | 0 | 0 | 0 | 1 | 1 | 1 | 1 | 8 |
| Wonch 2016 | 0 | 1 | 1 | 0.5 | 0 | 0 | 0.5 | 1 | 0 | 1 | 1 | 1 | 1 | 1 | 9 |
| Mao 2020 | 0 | 1 | 1 | 0 | 1 | 1 | 1 | 1 | 1 | 0 | 1 | 1 | 1 | 1 | 11 |
| McEwen 2012 | 0 | 1 | 0.5 | 0 | 1 | 0.5 | 0 | 1 | 0 | 0 | 1 | 1 | 1 | 1 | 8 |
| Sasaki 2020 | 0 | 1 | 1 | 0 | 1 | 1 | 0 | 0 | 1 | 0 | 1 | 1 | 1 | 1 | 9 |
| Rosa 2017 | 0.5 | 1 | 0.5 | 0.5 | 1 | 0.5 | 1 | 1 | 0 | 0 | 1 | 1 | 1 | 1 | 10 |
| Moses-Kolko 2011 | 0 | 1 | 0.5 | 0.5 | 1 | 0.5 | 0 | 1 | 0 | 1 | 1 | 1 | 1 | 1 | 9.5 |
| Dudin 2019 | 0 | 1 | 0.5 | 1 | 0 | 0 | 1 | 1 | 0 | 0 | 1 | 1 | 1 | 1 | 8.5 |
| Schnakenberg 2021 | 1 | 1 | 0 | 0 | 0 | 0.5 | 0 | 1 | 0 | 1 | 1 | 1 | 1 | 1 | 8.5 |
| Silverman 2007 | 0 | 1 | 1 | 0 | 1 | 1 | 0 | 0 | 1 | 1 | 1 | 1 | 1 | 1 | 10 |
| deRezende 2018 | 0 | 1 | 1 | 0.5 | 1 | 0 | 1 | 1 | 0 | 0 | 1 | 1 | 1 | 1 | 9.5 |
| Moses-Kolko 2012 | 0 | 1 | 1 | 0.5 | 1 | 0 | 0 | 0 | 0 | 0 | 1 | 1 | 1 | 1 | 7.5 |
| Ho 2017 | 0 | 0 | 0 | 0 | 0 | 0 | 0 | 1 | 0 | 1 | 1 | 1 | 1 | 1 | 6 |
| Li 2021 | 0 | 1 | 1 | 0 | 1 | 1 | 1 | 1 | 1 | 0 | 1 | 1 | 1 | 1 | 12 |
| Li 2021 | 0 | 1 | 1 | 0 | 1 | 1 | 1 | 1 | 1 | 1 | 1 | 1 | 0 | 1 | 11 |
| Zhang 2020 | 0 | 1 | 1 | 0.5 | 1 | 1 | 0.5 | 1 | 1 | 1 | 1 | 1 | 1 | 1 | 12 |
| Cheng 2022 | 0 | 1 | 0.5 | 1 | 1 | 1 | 1 | 1 | 1 | 1 | 1 | 1 | 1 | 1 | 12.5 |

Q = question
